# Current Hospital Topics

**Published:** 1906-05-19

**Authors:** 


### CURRENT HOSPITAL TOPICS.

### King's College Hospital.

The Elizabethan Fair and Fête in aid of this hospital will be held in Lincoln's Inn Hall and grounds from May 23 to 25 inclusive. King's College Hospital deserves well of the public because of the courage and wisdom displayed by the management in deciding to move the hospital from its present site to Dulwich. In passing, however, we may remark that one of the pressing needs of the new site appears, from the general block plan recently issued, to be more ground, as the superficial area of the present site is not sufficiently large to accommodate all the many buildings required for the purposes of the hospital and the school, if the buildings are to constitute a modern hospital of the best type. Here, then, is an opportunity for some wealthy man with a good business head and sound judgment to add to the new site before the new buildings are commenced. It is matter for regret that Miss Monk will not be able to superintend the furnishing and fitting of the new hospital buildings or to superintend the removal of the patients from Portugal Street to Dulwich. In order to provide an adequate hospital £300,000 it is estimated will be required, of which about half only has at present been raised. We know of no appeal which more deserves liberal and general support from Londoners than that of King's College Hospital. We hope, therefore, that the Elizabethan Fair and Fête may prove a record success, and it is certain to be if an attractive programme and much novelty of design and initiative in its arrangement can secure this result.

### London Hospitals and Assessment Committees.

The rating of hospitals in the Metropolis has long been a sore subject with the managers of many of them. This is the more surprising because twenty years ago the Committee of the Dreadnought Seamen's Hospital at Greenwich laid down a basis, which was accepted by the Assessment Committee, and which has made that institution one of the most equitably and smallest rated in London. After all, as one of the institutions of the district in which it is situated, a hospital must and does in fact tend materially to reduce the charges upon the poor rates, and is so entitled to the benevolent sympathy and help of the Assessment Committee. Unfortunately many hospitals appear to have regarded rating as a purely legal matter, and so to have left their appeals for reduction to their solicitors. In such circumstances the appeal becomes merely a technical matter, and is treated technically and broadly on the merits of the figures supplied by the overseers. The Greenwich plan was to interest the members of the rating authority in the work of the hospital; to bring to the notice of each one of them the history and character of the work done, the value of the treatment rendered to many patients relieved, who must otherwise come on the rates; and the claims to favourable consideration which those figures and facts generally warranted the governors in expecting at the hands of the Assessment Authorities. What can be done in this way towards reducing the rateable value of a hospital is well shown in the report of the Hospital for Epilepsy and Paralysis 1906. The rateable value was first assessed in 1902 at £1,667. On this a reduction was first obtained to £750, which was again reduced to £584. At that sum the rateable value stood until the quinquennial valuation took place in 1905. By the adoption of a similar system to that pursued at Greenwich the Committee are able to announce in their report that the rateable value has been reduced to £272. This victory for the hospital means a reduction of expenditure from £570 a year in rates to one of only £94. We congratulate Mr. H. Howgrave Graham, of whose great services the Committee record their appreciation, and all who have had any hand in securing this result. In 75 Metropolitan hospitals, large and small, containing 8,501 beds, with an expenditure of £917,000, the actual rates paid amount in the aggregate to £19,074. This is a heavy tax upon the resources of the voluntary hospital system, and it might be reduced by one-half or two-thirds if the managers of the higher rated hospitals especially would concert measures of a similar nature to those followed at Greenwich and Maida Vale when dealing with the assessment committee of their district. At the Royal London Ophthalmic Hospital, for instance, the annual rates amount to £1,008, or £7 per bed. At the Dreadnought Seamen's Hospital, however, a considerably larger hospital, covering a much more extensive site, the rates amount to only £195 a year, or something like £1 2s per bed. We might give a number of other instances, showing that certain hospitals have here a little gold mine, if they will only show business aptitude and pertinacity of purpose, combined with common sense and sound judgment when they move the assessment committee to reduce the present rateable value of their institutions. The successful management of a great hospital after all amounts in practice to the continuous exhibition of sound business judgment and an indomitable perseverance in securing adequate results, not only by treatment, but by the enforcement of economical principles in every department of the hospital world.

